# A Transcriptomics and Comparative Genomics Analysis Reveals Gene Families with a Role in Body Plan Complexity

**DOI:** 10.3389/fpls.2017.00869

**Published:** 2017-05-29

**Authors:** Eric M. Kramer, Wanying Li

**Affiliations:** Department of Physics, Bard College at Simon’s Rock, Great BarringtonMA, United States

**Keywords:** *Arabidopsis*, auxin, cell wall, development, *Physcomitrella*, receptor-like kinase, *Oryza sativa*, Affymetrix array

## Abstract

We analyzed tissue-specific transcriptomes of *Arabidopsis thaliana* and identified 66 gene families with a high frequency of “gradient genes” – genes showing a significant expression gradient between tissues. Gradient gene families include many with roles in hormone and peptide signaling, cell wall synthesis and remodeling, secondary metabolism, transcriptional regulation, and transport between cells. We compared the size of the gradient gene families among the genomes of four plant species with radically different body plans – a single-celled algae, a moss, a eudicot, and a monocot – and found that most of the gradient gene families (58/66) expanded in parallel with the evolution of morphological complexity. A novel measure of tissue diversity was used to show that members of any one gradient gene family tend not to be clustered in a single tissue, but are rather apportioned evenly across the tissues studied. Considered together, our results suggest that the diversification of these gene families supported the diversification of tissue types and the evolution of body plan complexity in plants.

## Introduction

The evolution of complex body-plans in vascular plants was made possible by the evolution of several innovations, including the ability to grow three-dimensional arrays of related cells, a competence for cell specialization, and positional signaling pathways to regulate these events (van den Berg et al., 1995; Graham et al., 2000; Harrison et al., 2009; Harrison, 2017).

**FIGURE 1:**
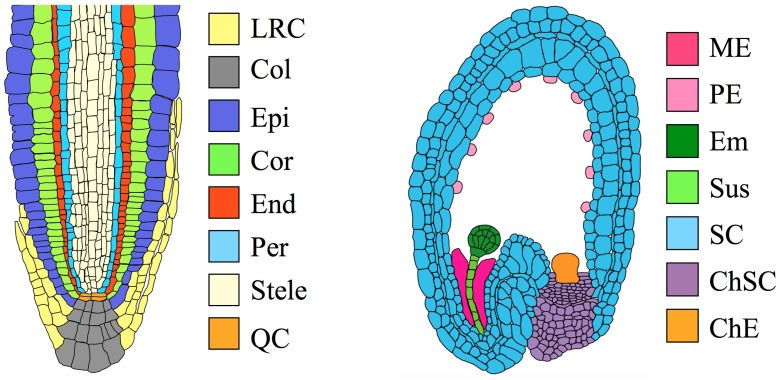
**Organs and tissues discussed in this paper. (Left)** Transverse section through an *Arabidopsis* root meristem. LRC, lateral root cap; Col, columella root cap; Epi, epidermis; Cor, cortex; End, endodermis; Per, pericycle; QC, quiescent center. The root has a maximum diameter of ∼130 μm (van der Weele et al., 2000). **(Right)** Transverse section through an immature *Arabidopsis* seed, showing an embryo at the globular stage. ME, Micropylar endosperm; PE, peripheral endosperm; Em, embryo; Sus, suspensor; SC, seed coat; ChSC, chalazal seed coat; ChE, chalazal endosperm. The seed has a maximum length of ∼500 μm (Western et al., 2000).

To illustrate how these innovations play a role in vascular plants, consider the *Arabidopsis thaliana* root (**Figure 1**). A cross-section through the root apex shows a series of concentrically arranged tissue layers (Dolan et al., 1993). Each layer is continuous around the central cylinder, and cell size and shape are tightly regulated. The geometry of these layers is initially established via cell divisions in the stem cell niche. However, van den Berg et al. (1995) used laser ablation to kill isolated cells in the root meristem, and subsequently observed neighboring cells undergo atypical divisions that restored the original pattern on a time scale of 1–4 days. These observations suggest that the identity of a cell is determined in part by information from neighboring cells. The importance of such positional information in the development of vascular plants is well-established (Furner and Pumfrey, 1992).

In addition to the signals that establish cell identity, there are other highly localized events essential for proper development. Many such events concern the synthesis, covalent modification, or remodeling of the cell wall. Lateral root emergence provides a well-studied example (Dubrovsky et al., 2008; Swarup et al., 2008; Kumpf et al., 2013; Yu et al., 2016). The lateral root begins as a primordium in the inner root, that subsequently penetrates the cell layers of the outer root and emerges into the growth medium. This process is carefully regulated via signals that pass from the primordium to the cells of the outer root (Swarup et al., 2008), and also via signals that pass between the cells of the outer root (Kumpf et al., 2013). These signals trigger cell wall remodeling and cell separation, to limit the damage that root emergence might otherwise cause. The separation of cells in the epidermis is typically limited to a single longitudinal radial wall, while contiguous walls remain intact. The mechanisms by which the plant achieves this degree of localization is unknown, although it is undoubtedly related to the wide diversity of cell-wall polysaccharides and hydrophobic modifications known to be maintained by cells of different type (Pattathil et al., 2010; Roppolo et al., 2011).

The two preceding examples illustrate how the evolution of vascular plants required the coincident evolution of mechanisms to localize developmental events to a single cell type, or even to a single cell wall. For this reason, it is a common practice for researchers to assign a putative role in development to gene families with a high frequency of tissue-specific members. Especially notable is Dinneny et al. (2008), who studied tissue-specific root transcriptomes in *Arabidopsis*. Their analysis identified 244 tissue-specific genes whose expression pattern is not altered by stress, and they speculated that many such genes play a role in cell identity.

In this paper we examine the possibility that many localized developmental events rely on a set of position-specific genetic modules, with paralogous modules functioning similarly across many or all tissue types. The evolution of a plant body with many different tissue types would therefore have occurred in parallel with the repeated duplication of these modules.

This idea receives circumstantial support from studies of leucine-rich repeat receptor-like kinases (LRR-RLKs). LRR-RLKs constitute a large, plant-specific family with established signaling functions in many aspects of development, including some of the localized events described above (DeYoung et al., 2006; Kumpf et al., 2013; Mandel et al., 2014). In an analysis preliminary to the research presented below, we discovered that LRR-RLKs are expressed in most plant tissues, and that each tissue expresses a unique combination of LRR-RLKs. The example provided by the LRR-RLK family inspired us to develop a quantitative approach to identify gene families with similar properties, and to examine whether these families also have an established or putative role in localized developmental events.

## Methods

### Transcriptome Atlases

We analyzed archived data sets from two groups of experiments (**Table 1**) that each collected tissue-specific transcriptomes in *Arabidopsis* using the Affymetrix gene chip ATH1 (Iyer-Pascuzzi et al., 2011; Belmonte et al., 2013).

**Table 1:** Data sets used in this paper.

Data set	Tissues	Reference
Roots of six dag seedlings; protoplasted and sorted using FACS	Columella root cap; lateral root cap and epidermis; cortex; endodermis; stele	Iyer-Pascuzzi et al., 2011 (archive)
Seeds with embryos at pre-globular stage; sampled using LCM	Pre-globular embryo (2–8 cells); micropylar endosperm; peripheral endosperm; seed coat	Belmonte et al., 2013 (archive)
Seeds with embryos at globular stage; sampled using LCM	Globular embryo; suspensor; micropylar endosperm; peripheral endosperm; seed coat	Belmonte et al., 2013 (archive)
Seeds with embryos at heart stage; sampled using LCM	Heart-stage embryo; micropylar endosperm; peripheral endosperm; seed coat	Belmonte et al., 2013 (archive)

*FACS, fluorescence-activated cell sorting; LCM, laser capture microdissection. The archive link connects to the raw data archived at EBI.*

Iyer-Pascuzzi et al. (2011) protoplasted roots of *Arabidopsis* seedlings expressing fluorescent markers for various cell types, and used fluorescence-activated cell sorting (FACS) to collect protoplasts from five tissues in the root apex (**Table 1**). Their paper examined the effects of several stresses on the root, but we only use their control sets (pH 5.7 and standard growth media).

Belmonte et al. (2013) used laser-capture microdissection to collect RNA from small regions of *Arabidopsis* seeds at a range of developmental stages. Since we are interested in meristematic and growing tissues, we limited consideration to seeds containing embryos at the pre-globular, globular, and heart-stages. After the heart stage, the seed coat stops expanding and enters a differentiation phase that we did not choose to include in the analysis (Western et al., 2000).

The transcriptomics data sets used in this paper are archived by EBI at www.ebi.ac.uk/arrayexpress (Brazma et al., 2003). Links to the EBI archive appear in **Table 1**.

### Vocabulary

**Table 1** shows the various data sets used in our analysis. We use the word *set* to refer to each of the four experiments: seeds at the pre-globular, globular, and heart embryo stages, and roots. We use the word *tissue* to describe each subset within a set, as shown. Note that a tissue may itself include several distinct cell types that were not distinguishable by the experimental technique used.

### Gradient Gene Families

For a detailed workflow describing the identification of gradient genes and gradient gene families, see the Supplementary Text.

### Genomes

We downloaded version 11 of the annotated genomes distributed by the Phytozome project (Goodstein et al., 2012). This includes genome version 5.5 for *Chlamydomonas reinhardtii*, version 3.3 for *Physcomitrella patens*, version TAIR10 for *A. thaliana*, and version 7 for *Oryza sativa* var *japonica*. The *Arabidopsis* and *Physcomitrella* genomes have been assembled into chromosomes, while the *Oryza* and *Chlamydomonas* genomes have not.

All genes in the Phytozome distribution have been annotated using Hidden Markov Models (HMM) of Pfam protein domains (Punta et al., 2012). In addition, we assessed all gene loci for predicted transmembrane domains using Phobius (Kall et al., 2004) and putative subcellular localizations using SUBAcon (Hooper et al., 2014).

Throughout this paper, only one gene per locus was used. The total number of unique gene loci for each species is 17,741 in *Chlamydomonas*, 32,926 in *Physcomitrella*, 27,416 in *Arabidopsis*, and 42,189 in *Oryza*.

## Results

### Finding the Gradient Gene Families

There are 100s of tissue- or organ-specific transcriptome data sets available in the EBI online archive (Brazma et al., 2003). However, we used three selection criteria to improve the quality and relevance of our results. First, we limited consideration to atlases that include either meristematic or embryonic tissues, as these are the most relevant for an analysis of development.

Second, we imposed strict limits on the replicate-to-replicate reproducibility of the sets, to minimize “noise” in the data that would tend to obscure differences in gene expression (Supplementary Methods).

Third, we only sought differentially expressed genes for adjacent sets of tissues from the same organ. We did not compare expression levels between different organs, or between different developmental stages of the same organ. This was done to focus results on localized developmental events.

**Table 1** shows the four data sets we selected for analysis, and **Figure 1** illustrates the size and location of each of the tissues. The data set of Iyer-Pascuzzi et al. (2011) includes five tissue types from seedling roots. The epidermis, cortex, and endodermis constitute concentric layers of cells that surround the stele, with the columella and lateral root cap located distally. The data sets of Belmonte et al. (2013) include several tissue types from immature seeds, including the embryo, suspensor, seed coat, and two regions of the endosperm. Readers seeking detailed reviews of the relevant anatomy are directed to Dolan et al. (1993) for the root and Goldberg et al. (1994) for the seed.

Our analysis of differential expression in these organs identified a total of 4858 genes with a significant expression gradient in at least one of the four sets. We call these “gradient genes.” The complete list of gradient genes may be found in Supplementary Table S1, along with annotations from TAIR10 and other sources.

Having compiled a list of gradient genes, we next examine which gene families are statistically over-represented. To assign genes to gene families, we rely on Phytozome project annotations (Goodstein et al., 2012), which use HMM to search for Pfam protein domains within each gene model (Punta et al., 2012).

Phytozome release 11 includes 5,046 Pfam domains with at least one occurrence in the *Arabidopsis* genome. However, as discussed in the section “Introduction,” we are interested in *large* gene families: those with enough members to play a putative role in the diversification of tissue types in vascular plants. Since we were examining 10 tissue types, we (somewhat arbitrarily) limited further analysis to gene families with 10 or more members. Of the 666 Pfam domain with at least 10 members in *Arabidopsis*, our analysis identified 88 domains as over-represented in the list of gradient genes (Supplementary Methods and Supplementary Table S2).

We curated the 88 Pfam domains by-hand into a smaller number of gene families using the procedures outlined in the legend of Supplementary Table S2. In part, this curation was done to avoid over-counting genes that share redundant Pfam domains. For example, the three multicopper oxidase Pfam domains occur together in 19 of 21 multicopper oxidase genes, and so are grouped together in a single gene family in the curated list. In addition, we parsed the kinase superfamily into three well-known subfamilies: soluble kinases, LRR-RLKs, and receptor-like kinases lacking an LRR domain (Supplementary Table S3). The resulting curated list contains 66 non-overlapping gradient gene families and is discussed more fully in the next section.

### The Gradient Gene Families

**Table 2** presents an overview of the gradient gene families, along with some descriptive comments. The table sorts the families by putative function, although for most genes on the list, function is inferred from family relationships and is yet to be characterized by experiment.

**Table 2:** Curated list of the gradient gene families.

Gene family	Comments	Localization	Expansion
**Cell division/cell cycle**			
Cyclins	Nuclear proteins that regulate the cell cycle	NUC	^∗^
Kinesins	Motor proteins with a role in cytokinesis	–	–
**Cell wall assembly/modification**			
Beta-galactosidase family	Carbohydrate metabolism; includes one member with a role in mucilage production.	APO	^∗^
CASP1 domain	Casparian strip formation	PM	^∗^
Cellulose synthase	–	PM	^∗^
Chitinase family	Some are stress response; some involved in cellulose synthesis	APO	^∗^
Expansins	Cell wall remodeling and cell growth	APO	^∗^
Extensins	Possible role as “scaffold” for cell wall (Cannon et al., 2008)	APO	^∗^
Fasciclin-like arabinogalactans	Possible roles in adhesion and/or signaling between cell and wall (Johnson et al., 2011)	–	–
Glycosyl hydrolase family 17	Carbohydrate metabolism; includes one member shown to degrade callose.	APO	^∗^
Glycosyl hydrolase family 9	Includes genes involved in cellulose metabolism	APO	^∗^
Haem peroxidase	Includes genes involved in lignin biosynthesis	APO	^∗^
Lipid transfer proteins (LTP)	Small, apoplastic proteins; uncharacterized function.	APO	^∗^
Pectin lyase-like superfamily	–	APO	^∗^
Pectin methylesterase/methylesterase inhibitor family	–	APO	^∗^
Pectinacetylesterase	–	APO/PM	^∗^
Trichome birefringence-like family	Includes proteins involved in cellulose synthesis	–	^∗^
Xyloglucan endo-transglycosylase (XET)	Cell wall remodeling and cell growth	APO	^∗^
**Chlorophyll**			
Light harvesting chlorophyll a/b binding protein (LHCB)	–	Plastid	–
**Peptidases**			
Eukaryotic aspartyl protease family	–	APO	^∗^
Papain family cysteine protease	Includes XCP1 and XCP2, with roles in autolysis during xylem formation (Avci et al., 2008)	–	–
Serine carboxypeptidase-like	Includes BRS1, involved in BR signalling	–	^∗^
Subtilisin-like serine peptidase	Includes SBT1.1, which cleaves the signaling peptide PSK4, and ARA12, involved in mucilage production	–	^∗^
**Secondary metabolism**			
3-Ketoacyl-CoA synthase family	Fatty acid elongation and wax biosynthesis	–	^∗^
Alcohol dehydrogenase	Includes proteins involved in lignin biosynthesis	CYT	–
Cytochrome p450s	Includes proteins involved in the metabolism of ABA, BR, GA, flavonoids, auxin, JA, and lignin	ER	^∗^
FAD linked oxidase	Includes proteins involved in cytokinin and BR metabolism	–	^∗^
GDSL lipases	Lipid metabolic activity; includes CDEF1, a cutinase	APO	^∗^
Gluconolactonase	Alkaloid synthesis	–	^∗^
Glutathione S-transferase	–	CYT	^∗^
HXXXD-type acyl-transferase	Some members involved in regulating BR levels or suberin biosynthesis	CYT	^∗^
Multicopper oxidase	Includes SKU5 and laccase families, some of the latter are involved in lignin biosynthesis	APO	^∗^
NAD(P)-binding Rossmann-fold superfamily	Some members involved in lignin biosynthesis	–	^∗^
NADPH respiratory burst oxidase	–	CYT	^∗^
Oxoglutarate/iron-dependent dioxygenase	Includes gibberellin oxidases and ACC oxidases	CYT	^∗^
S-adenosyl-L-methionine-dependent methyltransferase superfamily	Putative methyltransferases, mostly uncharacterized	–	^∗^
UDP-glycosyltransferase	Transfers activated glucose; a large family with roles in callose and lignin synthesis, and hormone conjugation (ABA, cytokinin, IBA, SA)	–	^∗^
**Signaling**			
Cyclic nucleotide gated channels (CNGC)	Ion channels gated by a range of ligands	–	–
IQ domain family	The IQ domain binds calmodulin, so these proteins have a role in calcium signaling	NUC	-
Lectins	Mostly kinases involved in stress responses	PM	^∗^
Leucine-rich repeat receptor-like kinases (LRR-RLKs)	Many have a role in plant pattern formation (e.g., BAM1, BAM2, ER, ERL2, GSO1, HSL2)	PM	^∗^
MIP1 domain family	Uncharacterized in plants; in other eukaryotes, MIP1/SIN1 regulates protein phosphorylation (Jacinto et al., 2006)	–	^∗^
Pollen Ole e 1 family	Function unknown; this family placed in the signaling category because the peptides are small, apoplastic, and developmentally regulated	APO	^∗^
Tetraspanins	Forms microdomains at the plasma membrane (Hemler, 2005); includes TORNADO2 (TRN2), which has several patterning defects (Cnops et al., 2006)	PM	^∗^
**Transcription factors and associated proteins**			
ARF and AUX/IAA families	Auxin response (e.g., ARF4, IAA24)	NUC	^∗^
Homeodomain family	Many roles in pattern formation; includes Homeobox-8 (HB-8), GLABRA 2 (GL2), REVOLUTA (REV), etc.	NUC	^∗^
LOB family	Involved in organ patterning; includes Asymmetric Leaves 2 (AS2) and JAGGED LATERAL ORGANS (JLO).	NUC	^∗^
WRKY family	Many involved with stress responses.	NUC	^∗^
**Transporters**			
ABC transporter family	Includes transporters of auxin, cutin, wax	PM	–
Amino acid permeases	Includes amino acid transporters and the LAX family of auxin influx carriers	PM	^∗^
Heavy metal transport/detoxification superfamily	Metal binding and transport	–	^∗^
Major facilitator superfamily	Includes sugar and nitrate transporters	–	^∗^
Major intrinsic protein (MIP)	Includes aquaporins, also includes transporters of boron, hydrogen peroxide, and small nitrogen compounds	–	^∗^
Multidrug and toxin efflux (MATE) family	Stress responses	PM	^∗^
Nodulin-like superfamily	Transporters with a wide range of substrates (Denance et al., 2014); includes SWEET sucrose transporters and WAT1 auxin transporters	PM	^∗^
PIN family	Auxin efflux carriers (e.g., PIN1)	PM	^∗^
**Uncharacterized**			
C1-like	–	–	^∗^
CYSTM domain	–	–	^∗^
Domain of unknown function DUF642	–	–	^∗^
Domain of unknown function DUF239	–	–	^∗^
FLZ domain	–	–	^∗^

*These gene families are over-represented among the genes exhibiting significant expression gradients. Functional information is derived largely from TAIR10 annotations, except where indicated with a citation. A family is given a localization label if at least 70% of its members share a common predicted localization according to the SUBAcon algorithm (Hooper et al., 2014). Location labels are APO, apoplast, CYT, cytoplasm, ER, endoplasmic reticulum, NUC, nucleus, PM, plasma membrane. Asterisks in the rightmost column indicate families with a significant expansion in gene counts in vascular plants (compared to the unicellular *Chlamydomonas*; see Supplementary Table S4 for more details). Hormone abbreviations: ABA, abscisic acid; BR, brassinosteroids; IBA, indolebutyric acid; JA, jasmonic acid; SA, salicylic acid.*

#### Cell Wall

Our analysis reveals a large number of families with a role in the cell wall, including enzymes responsible for synthesis and degradation of wall polysaccharides, covalent modifications like esterases, and the synthesis of hydrophobic components like cutin, wax, and lignin. In this category we have also included proteins that are themselves components of the cell wall, although in some cases their function remains obscure.

#### Cell Cycle

Our analysis finds the cyclin gene family, which regulates the cell cycle, and kinesins, which have a role in cell division.

#### Chlorophyll

The family of chlorophyll a/b binding proteins has no obvious relationship with the other families on the list, and is the only plastid-specific family. Xu et al. (2012) reports that this family has a role in abscisic acid and stress signaling, and Dingenen et al. (2016) reviews the evidence that plastids can regulate cell proliferation and expansion.

#### Peptidases

Peptidases are involved in protein processing and degradation, and some play a role in the processing of small signaling peptides that pass between cells. Very few peptidases have been functionally characterized in plants, despite the large size of these families.

#### Secondary Metabolism

Our analysis finds a large number of gene families with a role in secondary metabolism. Many of the gene families in this category have a putative or characterized role in the cell wall, but are placed in this functional category if some members have distinct functions. It is also notable that many of these families have a role in the synthesis or catabolism of plant hormones, including auxin, abscisic acid, brassinosteroids, cytokinin, gibberellins, and jasmonic acid (Davies, 2004). This result, that hormone-related genes tend to be differentially expressed between adjacent tissues, suggests that a complex network of spatially distinct hormone signals exists within developing organs. A previous analysis that examined transcriptomics data from hormone-treated whole seedlings found the interaction of plant hormones to be temporally complex (Nemhauser et al., 2006). Our analysis emphasizes the likely importance of spatial gradients in these interactions.

#### Signaling

The most important categories for our purposes are those gene families involved in signaling and transcriptional regulation. The family of LRR-RLKs is known to play a major role in numerous aspects of plant pattern formation, even though only a small fraction have been characterized in detail [e.g., ERECTA (Mandel et al., 2014)]. The lectin receptor kinases bind saccharides at the extracellular face of the PM and have putative roles monitoring the integrity of the cell wall (Gouget et al., 2006). The cyclic nucleotide gated channels transduce cytosolic signals into transmembrane ion currents, and have roles in stress responses and cell polarity (e.g., Gao et al., 2016), while the calmodulin-binding proteins have a putative role in calcium signaling. The roles of the other families on the signaling list are less well understood. Some tetraspanins are known to have a role in cell identity, as their loss-of-function results in dramatic changes to tissue patterning (Cnops et al., 2006). Two other families have been placed in this category for less direct reasons: the Olea extract 1 (Ole e 1) family has been included because it encodes small proteins known to be apoplastic and developmentally regulated (Hu et al., 2014), and the MADS-box interacting protein 1 (MIP1) family has been included because observations in other eukaryotes have revealed a signaling role (Jacinto et al., 2006).

#### Transcription

Our analysis identifies four families of transcription factors (TF), three of which have well-established roles in developmental gradients. The family of auxin response factors (ARFs), along with the co-TF family auxin/indole-3-acetic acid (AUX/IAA), mediates cell-specific auxin responses. The homeodomain superfamily includes many TFs known to have a role in cell identity (e.g., Masucci et al., 1996). The lateral organ boundary (LOB) family includes TFs that play a role in delineating distinct regions of a tissue or organ (e.g., Semiarti et al., 2001). The re-discovery of these TF families, along with the LRR-RLKs mentioned above, provides a check on the validity of our analysis and builds confidence that we have identified gene families with a role in development.

The fourth TF family on our list – WRKY – is a large family with diverse roles in stress response. In part, members of this family may have been induced by the techniques used to collect the transcriptome atlases – protoplasting roots in one case and the dissection of siliques in the other. However, whether the WRKY genes are induced or not, their presence among the gradient gene families highlights the fact that stress response pathways are often tissue-specific (Iyer-Pascuzzi et al., 2011).

#### Transporters

The transporter families in **Table 2** have a wide range of substrates, including metals, nitrogen compounds, and organic molecules. Especially notable is the fact that all major families of auxin transporters are represented on the list, including the PIN family of polar auxin efflux carriers.

In the following sections we characterize the gradient gene families as a group without reference to their individual roles.

### Gradient Gene Families and Body-Plan Complexity

To examine the putative role of the gradient gene families in morphological complexity, we compared their frequency in the genomes of four species: *C. reinhardtii*, a unicellular green algae, *P. patens*, a moss, *A. thaliana*, a eudicot, and *O. sativa*, a monocot. All four are model species in the Phytozome project (Goodstein et al., 2012), and their genomes have all been revised at least twice. *Chlamydomonas* is unicellular, but it should be remembered that it has a life cycle sophisticated enough to require several distinct cell types (Cavalier-Smith, 1974). *Physcomitrella* is a multicellular moss with a body plan that is simpler than *Arabidopsis* or *Oryza* (Sakakibara et al., 2003; Prigge and Bezanilla, 2010).

While attempts to quantify the relative complexity of multicellular organisms can involve some subjective judgments, the following features of *Physcomitrella* should be kept in mind. First, several *Physcomitrella* organs are one- or two-dimensional (Sakakibara et al., 2003). The filamentous caulonema, chloronema, and rhizoids all grow via the elongation of a single apical cell followed by the formation of new transverse cell walls behind the growing apex, while the leaf-like phyllids are a single cell thick. Second, *Physcomitrella* is a non-vascular plant, in the sense that it lacks specialized xylem or phloem tissue (Prigge and Bezanilla, 2010). Third, laser ablation experiments show that the cellular organization of the “bushy” gametophore of *Physcomitrella* is determined by cell lineage, with little evidence for the positional signals that regulate the development of vascular plants (Harrison et al., 2009). Thus, we argue that *Chlamydomonas*, *Physcomitrella*, and *Arabidopsis*/*Oryza* represent a gradient in both body plan complexity and the associated cell signaling.

**FIGURE 2:**
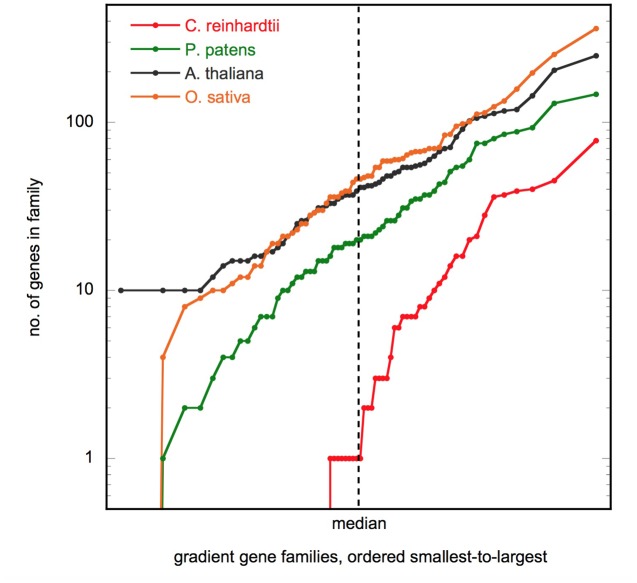
**Gene family size distribution.** Plot shows the number of genes in the 66 gradient gene families, ordered smallest to largest, for the four species shown. The axes are scaled so that a log-normal distribution will appear as a straight line. Figure made using KaleidaGraph v4.5 (Synergy Software).

If the gene families found in the previous section have a role in the evolution of body plan, then we would expect the number of genes per family to increase along this gradient. Indeed, this is the case (Supplementary Table S5). The median number of genes in the gradient families is 41 in *Arabidopsis* and 46 in *Oryza*. However, in *Physcomitrella*, the median is approximately half this (21), and in *Chlamydomonas* it is just 2. **Figure 2** shows the size distribution of the gene families. We see that the distribution of gene counts is approximately log-normal for the multicellular species, and that the medians are indeed representative of the overall differences.

It is notable that 25 of the 66 gradient gene families (38%) have no representatives in the unicellular algae *Chlamydomonas* (Supplementary Table S4). The gene families absent from *Chlamydomonas* are most prominent in the functional categories of cell wall metabolism (10 families) and signaling (4 families), and also include all 5 uncharacterized families. Conversely, with the exception of the extensin family, the gradient gene families are present in all three multicellular species.

As the reader might expect, the gradient gene families are not the only families that expand from *Chlamydomonas* to *Arabidopsis* and *Oryza*. However, we find that the expansion observed for the gradient gene families is still significant when compared to the larger set of all gene families (Supplementary Table S5). The median size of the gradient gene families is significantly larger than average in all three multicellular species, but not in *Chlamydomonas*. This is again consistent with the hypothesis that the gradient gene families have a role in morphological complexity.

One can also take a more fine-grained view and ask whether each individual family increases in size in parallel with body-plan complexity. This appears to be true for most of the gradient families. A Poisson distribution test that accounts for the effect of overall increases in genome size still finds that 91% (60/66) of gradient gene families are significantly larger in *Arabidopsis* than they are in *Chlamydomonas*, and 86% (57/66) are larger in *Oryza* than in *Chlamydomonas* (Supplementary Table S4). Using this measure, the moss *Physcomitrella* appears to be intermediate between algae and the vascular plants. 77% (51/66) of gradient gene families expand significantly between *Chlamydomonas* and *Physcomitrella*, and 80% (53/66) expand between *Physcomitrella* and *Arabidopsis* [the expansion between *Physcomitrella* and *Oryza* is 56% (37/66)]. This is again consistent with a positive correlation between the size of the gradient gene families and body-plan complexity.

### Localization of the Gradient Gene Families

#### Subcellular Localization

We used the SUBAcon database to estimate the subcellular localization of gene products in *Arabidopsis* (Hooper et al., 2014). SUBAcon is unusual among localization databases in that it uses published experimental observations to supplement sequence-based numerical predictions. However, the majority of protein localizations remain putative.

**FIGURE 3:**
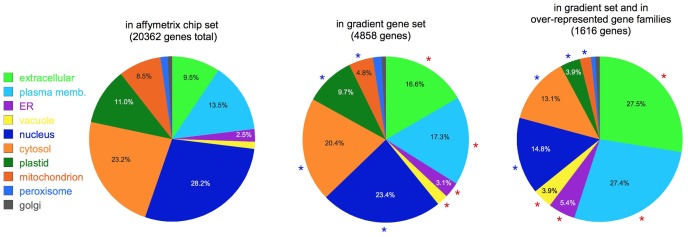
**Subcellular localization.** Pie charts show the putative localization of genes in the categories shown, as predicted by the SUBAcon algorithm (Hooper et al., 2014). Note the increased prominence of extracellular and plasma membrane localized genes in the gradient gene sets. Red asterisks: categories significantly over-represented compared to the whole Affymetrix set (*p* < 0.05); blue asterisks: significantly under-represented (*p* < 0.05). For clarity, percentages are not shown for categories with less than 2.5% of the total.

**Figure 3** shows a comparison of the putative localizations of gene products for three sets of genes: the set of all the genes in the Affymetrix chip, the set of gradient genes, and the subset of gradient genes that belong to the over-represented gene families (**Table 2**). As shown in the figure, comparisons between sets reveal significant changes in the proportion of most localization assignments (*p* < 0.05, hypergeometric test). Note, in particular, the changes in the relative proportion of extracellular and plasma membrane proteins. Together, they constitute 23% of the Affymetrix chip, but this fraction increases to 34% in the gradient gene set and 55% of the gradient gene family set. In other words, more than one third of the gradient genes and more than half the gradient family genes are predicted to function *outside* the cell. This is consistent with **Table 2**, which shows that many of the gene families have a role in either the cell wall or signaling at the plasma membrane.

#### Tissue Diversity

**FIGURE 4:**
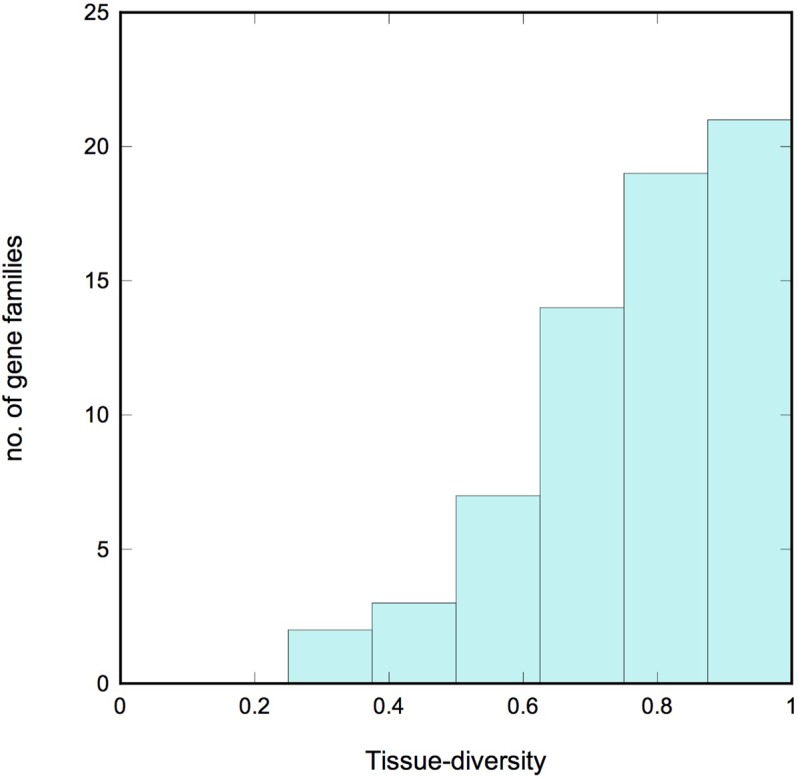
**Tissue diversity.** Histogram showing the tissue diversity of the gradient gene families. A value of 0 corresponds to a gene family whose expression is limited to a single tissue (“specialized”), while a value of 1 corresponds to a family whose members are expressed with equal frequency across all 10 tissues examined (“diverse”). Note the strong bias toward diversity.

We describe a gene family as *tissue-diverse* if its members are expressed across a broad range of tissues. To assess this property quantitatively, we used information theory to define a new measure of tissue-diversity. This measure is detailed in the Supplementary Methods section, but in brief it has a value of zero if all the members of a family occur only in a single tissue, and it reaches a maximum value of 1.0 if members of the family occur with equal frequency in all the tissues under consideration. Supplementary Table S4 lists the tissue-diversity value for each gradient gene family, and **Figure 4** shows a histogram of values. We see a strong bias toward tissue diversity, with a median value of 0.81. Only 5 of the 66 families have a tissue-diversity <0.5. These all appear to be expressed in just one or two tissues, and might therefore be described as “specialized.” Most of these specialized gene families are localized in the root tissue that includes epidermis and lateral root cap cells.

## Discussion

As discussed in the Introduction, one of the inspirations for this paper was the large gene family of LRR-RLKs, which includes many members with established roles in localized developmental events (DeYoung et al., 2006; Kumpf et al., 2013; Mandel et al., 2014). The LRR-RLKs are heavily represented among the gradient genes. Notably, the LRR-RLKs expand significantly along the body-plan complexity gradient. The unicellular algae *Chlamydomonas* has no LRR-RLKs, but the number increases to 130 in the moss *Physcomitrella*, and increases again to 204 and 254, respectively, in the vascular plants *Arabidopsis* and *Oryza*. All of these increases are statistically significant, even after accounting for the general increase in genome size between species. We also introduced a new measure of tissue diversity (Supplementary Methods) that ranges from a low of 0 for a gene family expressed in a single tissue to a high of 1.0 for a gene family with members distributed evenly amongst the various tissues examined. The value of this measure for the LRR-RLKs, for the 10 tissues we consider here, is 0.89. In short, LRR-RLKs excel at all the measures we consider in this paper, which gives us confidence that the properties considered here are correlated with a role in development.

The LRR-RLKs are just one family among the 66 gradient gene families identified in our analysis, and they share similar features. Most gradient gene families (58/66) show evidence of substantial expansion during the evolution of complex body plans. A comparison of the unicellular algae *Chlamydomonas*, the moss *Physcomitrella*, the eudicot *Arabidopsis*, and the monocot *Oryza* suggests that the gradient gene families increased in size by a factor of ∼10 during the evolution of multicellularity, and doubled again during the evolution of vascular plants. We also find that gradient gene families tend to be tissue-diverse. That is, their members tend to be distributed among most of the tissues examined, rather than specialized to just one or two tissue types. This is consistent with the hypothesis that most gradient gene families diversified in parallel with the diversification of tissue type.

Many of the gradient gene families identified by our analysis are known to play functionally important roles in the organization of complex body plans. In addition to the LRR-RLKs (DeYoung et al., 2006), (Kumpf et al., 2013), (Mandel et al., 2014), homeobox and LOB TF (Masucci et al., 1996; Semiarti et al., 2001), and the tetraspanins (Cnops et al., 2006) have well-established roles. In addition to these, our analysis finds several families involved in plant hormone metabolism and signaling (Davies, 2004). The plant hormone auxin appears prominently in the list of gradient gene families, which includes the PIN family of transporters, the WALLS ARE THIN 1 (WAT1) family of transporters (Ranocha et al., 2013), and the ARF family of TF. Many other plant hormones also appear in the TAIR10 annotations of gradient gene families. In addition to auxin, we find that gibberellins, brassinosteroids, jasmonic acid, and salicylic acid are all over-represented in the annotations (*p* < 0.05 using a hypergeometric test; results in Supplementary Table S6). Of these, auxin and brassinosteroids have the most direct influence on body-plan (Okada et al., 1991; Gendron et al., 2012). It is perhaps significant that these two hormones appear with the highest frequency in the gradient family set.

The largest category in the gradient gene family list includes those families with a role in the cell wall. This is immediately apparent from **Table 2**, which includes 16 families with a principal role in cell wall synthesis, metabolism, integrity, or modification, and an additional seven families that play some role in the secondary metabolism of wax, lignin, cutin, or suberin. In fact, approximately one third of all wall-related genes on the Affymetrix chip appear in our gradient gene family list (Supplementary Table S6). One likely explanation is that our gradient gene analysis focused on the transcriptomes of root meristems and growing seeds, both of which are actively producing primary and secondary cell walls. Thus, the expression of cell-wall related genes will be high, and differential expression patterns will be relatively easy for our algorithm to detect. The complex pattern of cell wall gene expression is reflected in the complexity of the walls themselves. Monoclonal antibody techniques reveal a surprising degree of spatial variation among the polysaccharides of the cell wall (Pattathil et al., 2010), and the highly localized nature of hydrophobic barriers in the root is well-known (Roppolo et al., 2011).

Additional support for our analysis comes from the results of Pattison et al. (2015), who use laser-capture microdissection and RNA-sequencing to examine tissue-specific transcriptomes in developing fruits of currant tomato (*Solanum pimpinellifolium*). Their Figure 6 identifies functional annotations over-represented among genes with tissue-specific expression patterns. The list of annotations with *p* < 0.01 shows substantial overlap with those listed in **Table 2**, including cell wall metabolism, hormone-related functions, kinases, proteases, TF, and transporters. Thus, an independent analysis in a different eudicot finds categories similar to those found here.

Many of the gradient gene families are yet to be adequately characterized. Tetraspanins have a role in signaling at the plasma membrane (Cnops et al., 2006), but the functional details require additional clarification. Lipid transfer proteins show a remarkable ability to spatially segregate in response to changes in the geometry of the cell wall (Ambrose et al., 2013), although the means by which this is accomplished, and possible developmental consequences, are unknown. The MIP1 domain plays a role regulating phosphorylation in eukaryotes (Jacinto et al., 2006), but their role in plants is yet to be examined. Lastly, five gene families in **Table 2** have no established function in any organism. These five families are notably absent from the unicellular algae *Chlamydomonas*, but present in the multicellular species examined. We therefore suggests that at least some of these have a role in the development of multicellular plants. We hope our analysis will encourage researchers to explore these families in more detail.

## Author Contributions

WL conducted preliminary analyses and participated in early planning discussions. EK planned and conducted the analyses, and wrote the paper.

## Conflict of Interest Statement

The authors declare that the research was conducted in the absence of any commercial or financial relationships that could be construed as a potential conflict of interest.
