# Supplementary material for: A Transcriptomics and Comparative Genomics Analysis Reveals Gene Families with a Role in Body Plan Complexity
Source: Front Plant Sci. 2017 May 29;8:869. doi: 10.3389/fpls.2017.00869 (PMC5446989; doi:10.3389/fpls.2017.00869)
Supplement: Supplementary file 3 [file Supplementary_Text.pdf]

Supplemental Text for "A transcriptomics and comparative genomics analysis reveals gene families with a role in body plan complexity"

Supplemental Methods

Our workflow to identify gradient genes and gradient gene families is summarized in Fig. S1. Additional details are given in the text below.

Gene annotations. We used an updated list of gene assignments for the ATH-1 chip, current to TAIR10 (ATH1-121501 Annotations, Release 35, from Affymetrix). We used only one probe per gene, and we avoided the use of probes that match more than one gene. Excluding pseudogenes, transposable elements, tRNA, and miRNA leaves 20362 unique gene loci on the ATH-1 chip.

Normalization. Using Expression Console v1.4.1.46 (Affymetrix), we recalculated all signals starting from the archived raw data sets (.CEL files) using Robust Multichip Average (RMA) normalization (Gohlmann and Talloen, 2009).

Replicate quality. We limited our analysis to the four data sets shown in Table 1 because they all satisfy stringent criteria on replicate reproducibility. They have a replicate-replicate correlation coefficient, $R > 0.9$, and individual gene signals tend to vary by less than 2 units on the RMA scale from one replicate to the next (>90% of the genes satisfy this condition).

Differentially expressed genes were identified using the workflow shown in Supplemental Figure 1 (Iyer-Pascuzzi et al., 2011). The three pieces of software used were Excel for Mac v15 (Microsoft), Kaleidagraph v4.5 (Synergy), and a custom FORTRAN program gradient, included as supplemental files.

Limiting false positives. We conducted pairwise comparisons between the tissues of each set shown in Table 1, but not between sets. We also did not compare tissues from seeds at different developmental stages. As shown in Table 1, we used two data sets with 5 tissues (10 pairwise comparison each) and two data sets with 4 tissues (6 pairwise comparisons each). Thus, there were a total of 32 total pairwise comparisons in our analysis.

The threshold for differential expression is $p < 10^{-4}$. With 20,362 unique genes in the Affymetrix set, this gives an estimated $10^{-4} \times 20362 = 2.03$ false positives per

pairwise comparison. Since there are a total of 32 pairwise comparisons, that gives an estimated ~65 false positives. The total list of differentially expressed genes – that is genes found to be differentially expressed in at least one pairwise comparison – contains 4858 genes, for a false positive rate of approximately 64/4858 = 1.3%.

Gradient gene families. Having compiled a list of gradient genes, we next examined which gene families are statistically over-represented. To assign genes to gene families, we relied on Phytozome project annotations (Goodstein et al., 2012), which use Hidden Markov Models (HMM) to search for Pfam protein domains within each gene model (Punta et al., 2012).

Phytozome release 11 finds 5,046 Pfam domains with at least one occurrence in the *Arabidopsis* genome. However, as discussed in the Introduction, we are interested in *large* gene families: those with enough members to play a putative role in the diversification of tissue types in vascular plants. Since we were examining ten tissue types, we (somewhat arbitrarily) limited further analysis to gene families with ten or more members. There are 666 such families. We next used the hypergeometric test (Selvin, 1998) to assess whether each Pfam domain was over-represented in our gradient gene set and the technique of (Benjamini and Hochberg, 1995) to limit the false discovery rate (FDR) to 5%.

Tissue-Diversity is a new measure to quantify the degree to which a gene family is distributed evenly amongst the tissues under consideration. We started by assigning a tissue localization to each gradient gene. If the gene was differentially expressed among the tissues of the root set, then it was assigned only to that tissue in which its RMA signal was largest. Similarly for each of the three seed sets. To avoid the over-counting that might result from our use of three different developmental stages in the seed, a gene present in more than one developmental stage of the same tissue (e.g. the embryo) was only counted towards that tissue once, even if it was present at all three time points. Each gene in the gradient list was thereby assigned a localization to at least one of the ten tissues in Table 1.

To define our measure of tissue diversity, we borrowed a result from information theory that is more commonly used to describe the information content

of letters or words in a message (Shannon, 1948). The ten tissue types compose an alphabet of ten "letters". Each gene family is then represented by a "word" composed of the letter assignments for each gene in the family. For example, a gene family with exactly three genes in the gradient set - two in the root epidermis and one in the root cortex - would be assigned the word "EEC". One can then calculate the number of bits required to communicate that word. This is called the Shannon information (Shannon, 1948). If a word is composed of N different letters, each occurring with frequency $0 \leq f_i \leq 1$, then the Shannon information is

$$H = \sum_{i=1}^{N} f_i \log_2(f_i)$$

Illustrative examples: A gene family with five members, all with high and significant expression in the root epidermis, would be represented by the word "EEEEE". The frequency of the character E is $f_E = 1$, and the rest of the letters occur with a frequency of $f_i = 0$. The information content is then $H = 0$. Another gene family with five members – two with high expression in the epidermis, two with high expression in the cortex, and one that is not differentially expressed – would be represented by the word "EECC". In this case, $f_C = 0.5$, $f_E = 0.5$, and the rest $f_i = 0$. The information content is then $H = 1$. Gene families with members in more tissues will tend to have a larger H, up to the hypothetical maximum of $\log_2(N)$.

To define the tissue diversity of a gene family, we divided the information content of the "word" by the hypothetical maximum value of $\log_2(10)$. The tissue diversity, defined as $H/ \log_2(10)$, then interpolates between a value of 0 for a gene family that only occurs in one tissue type and a value of 1 for a gene family that occurs with equal frequency in all ten tissue types (maximal tissue diversity).

Supplemental Figure Legends

Figure S1. Summary of the workflow used to identify gradient genes and gradient gene families.

Supplemental Table Legends

Table S1. List of the 4,858 gradient genes. Data columns, reading left to right: locus accession number, link to TAIR website, ATH1 chip ID number, Pfam domains predicted by Phytozome (Goodstein et al., 2012), number of transmembrane domains predicted by Phobius (Kall et al., 2004), family assignments and annotations from TAIR10, and putative localization predictions from SUBAcon (Hooper et al., 2014).

Table S2. The 88 Pfam domains over-represented among gradient genes, and their curation into 66 gene families. Data columns, reading left to right: Pfam ID, Interpro domain name, name of corresponding curated gene family (as listed in Table 2), Pfam ID for curated family (as listed in Table S4). Color-coded groups are arranged to show Pfam domains that often occur together in the same protein. Excepting the protein kinase superfamily, redundant Pfam categories were eliminated from the curated list if they overlapped with a larger Pfam category. The protein kinase superfamily was parsed into three groups. The Phobius transmembrane predictor (Kall et al., 2004) was used to sort kinases into those having or lacking a transmembrane domain. Kinases lacking a TM domain were grouped as soluble kinases. Those having a TM domain were grouped as receptor-like kinases, and divided further into those with or without a leucine-rich repeat (LRR) domain (Table S3 shows this parsing of the kinases). Of the three groups, only the LRR receptor-like kinases (LRR-RLKs) were significantly over-represented in

the gradient gene set (p = 9.7x10^{-14}). The lectins were left as a distinct gene family because not all lectins have a kinase domain.

Table S3. Kinases on the Affymetrix ATH1 chip, organized into three categories. Data columns same as Table S1. Leucine-rich repeat receptor-like kinases (LRR-RLKs; green), other receptor-like kinases (white), and soluble kinases (orange). Kinase domain assignments include Pfam domains PF07714 and PF00069. LRR domain assignments include Pfam domains PF00560, PF08263, and PF13855. Trans-membrane domains are assigned using Phobius (Kall et al., 2004).

Table S4. Curated list of the gene families over-represented in the gradient gene list, including localization data and comparisons across species. Data columns, reading left to right: Pfam ID, gene family name (functional categories in bold), family size in *Chlamydomonas*, *Physcomitrella*, *Arabidopsis*, and *Oryza* (blue highlights 0 values), the statistical significance of family expansion among the various species (p-values calculated using the Poisson distribution; orange highlights those p-values that satisfy the false-discovery rate threshold < 0.05, calculated using the technique of (Benjamini and Hochberg, 1995)), Pfam ID and family name (repeated for convenience), gene counts in the gradient gene list and the ATH1 chip, gene counts in each of the ten tissues considered in this paper, the Shannon information and the tissue-diversity (for discussion of the last two columns, see Supplemental Methods).

Table S5. To assess the significance of the median family size for the 88 Pfam categories over-represented in the gradient gene set, we compared the median to the median size of all 666 gene families in *Arabidopsis* with 10 or more members.

Illustrative example: the gradient gene families in *Arabidopsis* have a median size of 41 members. Among the 666 large gene families, 122 have a size of 41 or larger. We use the hypergeometric distribution to calculate the probability of randomly drawing from the full set of 666 families a subset of 88 families, at least 44

of which have a size of 41 or larger. In Excel notation, this probability is 1-HYPGEOM.DIST(44-1,88,122,666,TRUE).

Medians significantly larger than expected for that species ($p<0.05$) are indicated with orange highlighting.

Table S6. Frequency of hormone and wall-related keywords in the annotation of all genes on the ATH1 chip, and in the set of gradient genes that are also members of the gradient gene families. Orange indicates over-represented keywords ($p < 0.05$; hypergeometric test).

References

Benjamini, Y., and Hochberg, Y. (1995). Controlling the false discovery rate: a practical and powerful approach to multiple testing. *Journal of the Royal Statistical Society. Series B* 57**,** 289-300.

Gohlmann, H., and Talloen, W. (2009). *Gene expression studies using Affymetrix microarrays.* New York: CRC Press.

Goodstein, D.M., Shu, S., Howson, R., Neupane, R., Hayes, R.D., Fazo, J., et al. (2012). Phytozome: a comparative platform for green plant genomics. *Nucleic Acids Research* 40**,** D1178-D1186.

Hooper, C.M., Tanz, S.K., Castleden, I.R., Vacher, M.A., Small, I.D., and Millar, A.H. (2014). SUBAcon: a consensus algorithm for unifying the subcellular localization data of the *Arabidopsis* proteome. *Bioinformatics* 30**,** 3356-3364.

Iyer-Pascuzzi, A.S., Jackson, T., Cui, H., Petricka, J.J., Busch, W., Tsukagoshi, H., et al. (2011). Cell identity regulators link development and stress responses in the *Arabidopsis* root. *Developmental Cell* 21**,** 770-782.

Kall, L., Krogh, A., and Sonnhammer, E.L.L. (2004). A combined transmembrane topology and signal peptide prediction method. *Journal of Molecular Biology* 338**,** 1027-1036.

Punta, M., Coggill, P.C., Eberhardt, R.Y., Mistry, J., Tate, J., Boursnell, C., et al. (2012). The Pfam protein families database. *Nucleic Acids Research* 40**,** D290-D301.

Selvin, S. (1998). *Modern Applied Biostatistical Methods.* Oxford University Press.

Shannon, C.E. (1948). A mathematical theory of communication. *The Bell System Technical Journal* 27**,** 379-423,623-656.
