# Supplementary figures and images for: A Transcriptomics and Comparative Genomics Analysis Reveals Gene Families with a Role in Body Plan Complexity
Source: Front Plant Sci. 2017 May 29;8:869. doi: 10.3389/fpls.2017.00869 (PMC5446989; doi:10.3389/fpls.2017.00869)

Figure S1. Gradient gene families workflow

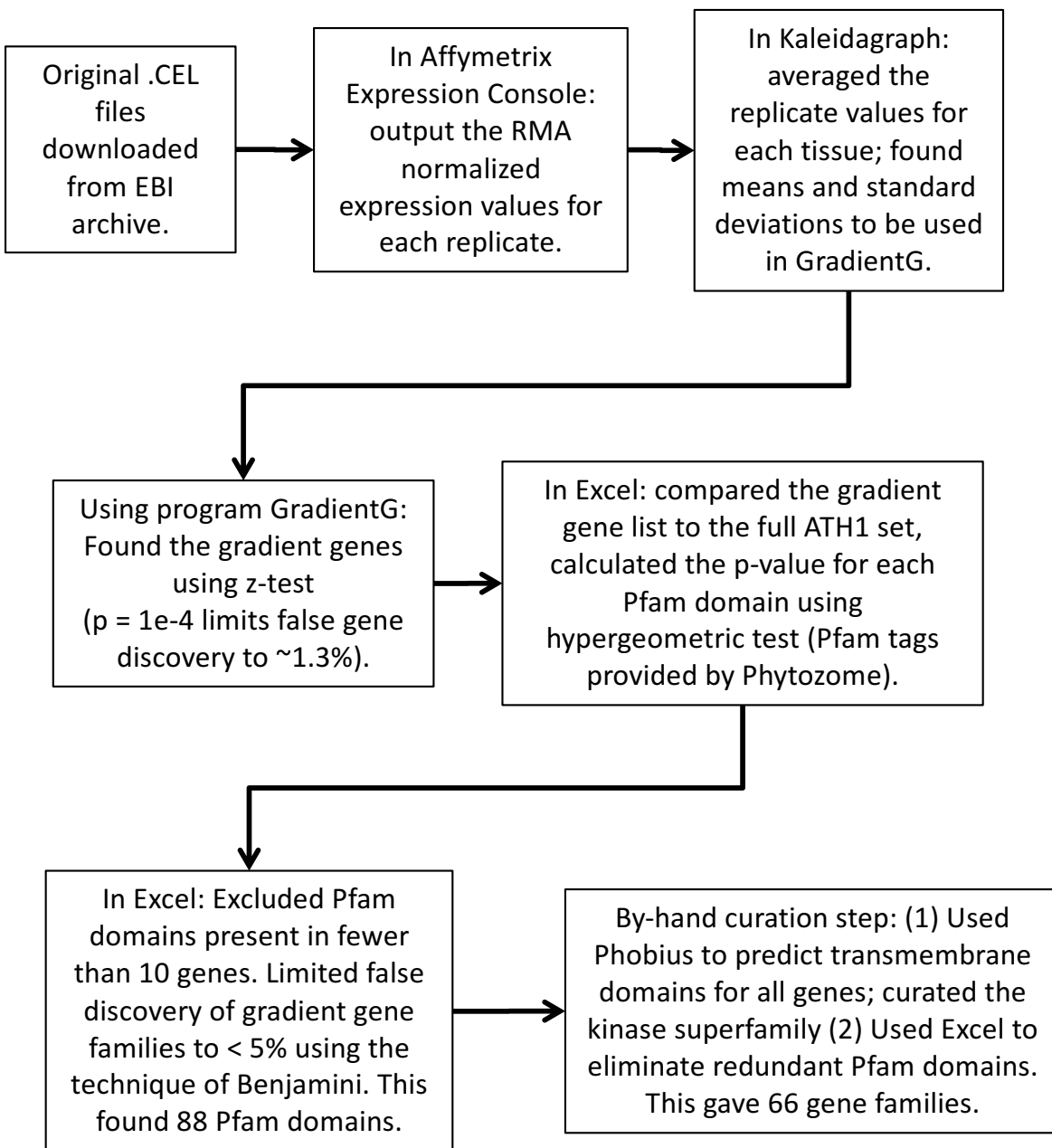

Supplement: Supplementary file 4 [file Figure_S1.pdf]
